# Supplementary material for: Sepsis-like Energy Deficit Is Not Sufficient to Induce Early Muscle Fiber Atrophy and Mitochondrial Dysfunction in a Murine Sepsis Model
Source: Biology (Basel). 2023 Mar 30;12(4):529. doi: 10.3390/biology12040529 (PMC10136327; doi:10.3390/biology12040529)
Supplement: Supplementary file 1 [file biology-12-00529-s001.zip › biology-2147364-supplementary.pdf]

Table S1. Description of the chronological period according to the recommendations of the European Society for Clinical Nutrition and Metabolism (ESPEN) (Singer *et al.* Clinical Nutrition 2019, https://doi.org/10.1016/j.clnu.2018.08.037) and its correspondence in our murine study.

Clinical human study				Preclinical murine study	
ESPEN designation		Time	Physiological relevance	Correspondence	Criteria
**Acute phase**	**Early**	Day 1 to 2	Catabolism	**Day 1**	**Before the first death**
	Late	Day 3 to 7		Day 2 to 5	
Convalescence or chronicity		From day 8	Anabolism, Post-Intensive Care Syndrome or Persistent Inflammation, Immunosuppression and Catabolism Syndrome	From day 6	After the last death and the 5-day resuscitation

The timing of our study is referred to in blue and bold.

Table S2. Description of the Murine Sepsis Score. According to Shrum *et al.* BMC Res Notes 2014 (DOI: 10.1186/1756-0500-7-233).

Variable	Score and description
Appearance	0- Coat is smooth.
	1- Patches of hair piloerected.
	2- Majority of back is piloerected.
	3- Piloerection may or may not be present, mouse appears "puffy".
	4- Piloerection may or may not be present, mouse appears emaciated.
Level of consciousness	0- Mouse is active.
	1- Mouse is active but avoids standing upright.
	2- Mouse activity is noticeably slowed. The mouse is still ambulant.
	3- Activity is impaired. Mouse only moves when provoked, movements have a tremor.
	4- Activity severely impaired. Mouse remains stationary when provoked, with possible tremor.
Activity	0- Normal amount of activity. Mouse is any of: eating, drinking, climbing, running, fighting.
	1- Slightly suppressed activity. Mouse is moving around bottom of cage.
	2- Suppressed activity. Mouse is stationary with occasional investigative movements.
	3- No activity. Mouse is stationary.
	4- No activity. Mouse experiencing tremors, particularly in the hind legs.
Response to stimulus	0- Mouse responds immediately to auditory stimulus or touch.
	1- Slow or no response to auditory stimulus; strong response to touch (moves to escape).
	2- No response to auditory stimulus; moderate response to touch (moves a few steps).
	3- No response to auditory stimulus; mild response to touch (no locomotion).
	4- No response to auditory stimulus. Little or no response to touch. Cannot right itself if pushed over.
Eyes	0- Open.
	1- Eyes not fully open, possibly with secretions.
	2- Eyes at least half closed, possibly with secretions.
	3- Eyes half closed or more, possibly with secretions.
	4- Eyes closed or milky.
Respiration rate	0- Normal, rapid mouse respiration.
	1- Slightly decreased respiration (rate not quantifiable by eye).
	2- Moderately reduced respiration (rate at the upper range of quantifying by eye).
	3- Severely reduced respiration (rate easily countable by eye, 0.5 s between breaths).
	4- Extremely reduced respiration (>1 s between breaths).
Respiration quality	0- Normal.
	1- Brief periods of laboured breathing.
	2- Laboured, no gasping.
	3- Laboured with intermittent gasps.
	4- Gasping.

Table S3. Description of the antibodies used in Western-blot and Immunofluorescence experiments.

**Primary antibodies**		
GAPDH	#2118S Cell Signaling	1/ 5000
Mfn2	#9482S Cell Signaling	1/ 1000
Drp1	ab184247 Abcam	1/ 1000
Pgc1α	ab191838 Abcam	1/ 500
OXPHOS rodent cocktail	ab110413 Abcam	1/ 1000
Pink1	BC100-494 Novus	1/ 1000
phospho-Ser65 Parkin	ab154995 Abcam	1/ 1000
p62 (sequestosome 1)	#5114 Cell Signaling	1/ 1000
LC3B	Ab51520 Abcam	1/ 1000
Laminin-α2 (4H8-2)	Sc-59854 Santa Cruz	1/ 100
VDAC1/3	Ab14734 Abcam	1/ 1000
**Secondary antibodies**		
Mouse IgG	#7076S Cell Signaling	1/ 4000
Rabbit IgG	#7074S Cell Signaling	1/ 4000
Rat IgG conjugated AlexaFluor 555	A21434 Invitrogen	1/ 500

Table S4. Description of the primers used in RTqPCR experiments.

Gene	Sequence: forward	Sequence: reverse	Accession number
**Relative RNA expression**			
Ppia	GCGTCTCCTTCGAGCTGTTT	GCGTGTAAAGTCACCACCCT	NM_008907.2
Tfam	ACCGTATTGCGTGAGACGAA	CAGACAAGACTGATAGACGAGGG	NM_009360.4
Sirt1	GCCGCGGATAGGTCCAT	GCTTTGGTGGTTCTGAAAGGA	NM_019812.3
Nrf1	TGCATCTCACCCTCCAAACC	GAAGCTGAGCCTGGGTCATT	NM_001164226.1
Pgc1α	CGGAAATCATATCCAACCAG	TGAGGACCGCTAGCAAGTTTG	NM_008904.3
Bnip3	GCACTCTGTCTGAGGAAGATTAT	TGAGAGTAGCTGTGCGCTTC	NM_009760.4
Ulk1	TCCCTACACACCTTCTCCCC	AGCCAACAGGGTCAGCAAAT	NM_001347394.1
Atg5	GGAGAGAAGAGGAGCCAGGT	GCTGGGGGACAATGCTAATA	NM_053069.6
Atg7	GCCTAACACAGATGCTGCAA	TGCTCTTAAACCGAGGCTGT	NM_028835.5
Atg8l	GAGGACCACCCCTTCGAGTA	GTGGGAGGGATGGTGTTGTT	NM_020590.4
Atg12	TAAACTGGTGGCCTCGGAAC	ATCCCCATGCCTGGGATTTG	NM_026217.3
Beclin1	CCGCGGTAGAACGAGCC	AAGTAATGGAGCTGTGAGTTCCT	NM_019584.4
Mfn1	GCACAGAGGGTGCTGCTCGG	TGGGCTGCATTATCCGGGGC	NM_024200.5
Mfn2	GGGGCCTACATCCAAGAGAG	GCAGAACTTTGTCCCAGAGC	NM_133201.3
Opa1	GATGACACGCTCTCCAGTGAAG	CTCGGGGCTAACAGTACAACC	NM_133752.4
Drp1	CGGTTCCCTAAACTTCACGA	GCACCATTTCATTTGTCACG	NM_152816.4
Fis1	CCGGCTCAAGGAATATGAAA	CCATGCCTACCAGTCCATCT	NM_025562.3
MAFbx	GGGGTCACCCTGCAGCTTTGC	GGGGAAAGTGAGACGGAGCAGC	NM_026346.2
Mu-RF1	ATGGACCGGCACGGGGTGTA	GCACATCGGGTGGCTGCCTT	NM_001039048
Ubc	CGCGCTGATCCCTCCG	CTGCATCGTCTCTCTCACGG	NM_019639.4
Foxo1	CATCCACTCGTAGATCTGCG	TCGTCGCGCCGCAACGCGTG	NM_019739
Foxo3a	CCGGACAAACGGCTCACTT	GGTTGTGCCGGATGGAGTT	NM_019740.3
Redd1	TCTCGAACTCCGGCCGCTGA	CCCAATCGCGCCTGGGACAG	NM_029083
Bcat2	CCCTCCTGGCGGACCCTTCAT	AGCTGGTGGTCTGGCCCGT	NM_009737
Klf15	AAGCCCTTTGCCTGCACCTGG	CGTACTGCGCGGCTGCTTCG	NM_023184
**Mitochondrial DNA copy number**			
Ppia	ACACGCCATAATGGCACTGG	CAGTCTTGGCAGTGCAGAT	NM_008907.2
Nd1	TCCGAGCATCTTATCCACGC	GTATGGTGGTACTCCCGCTG	NC_005089.1
Nd2	ATCCTCCTGGCCATCGTACT	ATCAGAAGTGGAATGGGGCG	NC_005089.1

Table S5. Summary of the impact of extracellular hydration state on body composition and muscle parameters.

	Influence by hydration state	SPF mice	Sepsis mice
**Body weight**	Yes	↓	↔
**Free fluid mass**	Yes	↓ Extracellular dehydration	↑ Extracellular hyperhydration
**Fat mass**	No	↓	↓
**Lean mass**	Yes	↓ Extracellular dehydration	↔ Extracellular hyperhydration
**Wet weight of hind limb muscles**	Yes	↓ Extracellular dehydration	↓ Extracellular hyperhydration
**TA cross-sectional area or Feret's diameter**	No	↔	↓

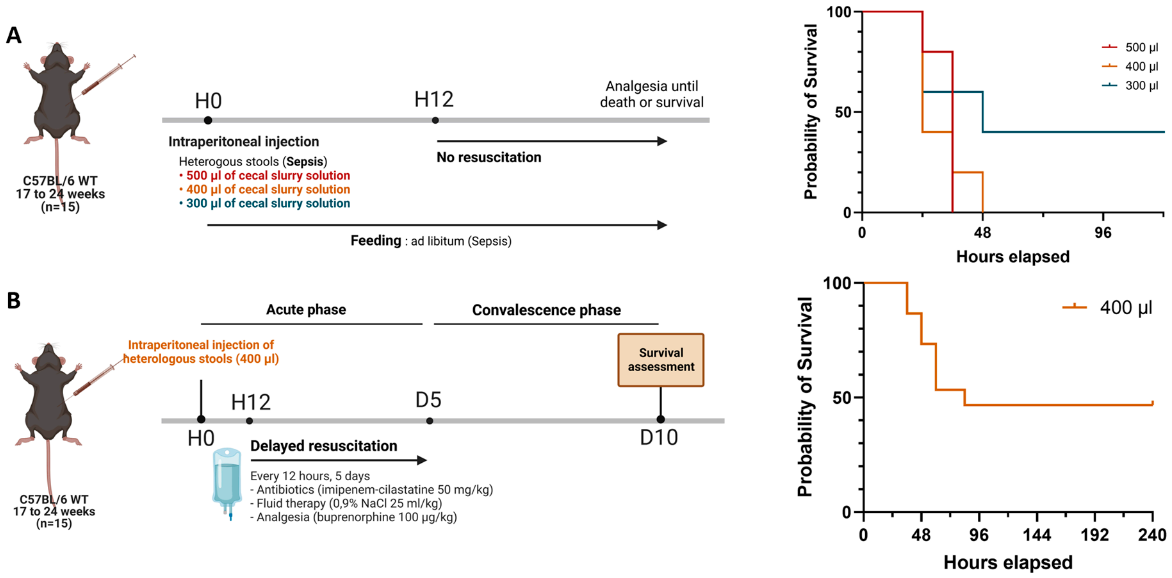

Supplementary Figure S1. Characteristics of the cecal slurry injection model. (A) Survival experiment without resuscitation: 5 mice per group were injected with three doses of cecal slurry solution (500 µl in red, 400 µl in orange, and 300 µl in blue) and were not resuscitated. All the mice died at 400 µl determining the minimal lethal dose. **(B)** Survival experiment with resuscitation: 15 mice were injected with 400 µl of cecal slurry solution and were resuscitated 12 hours after the insult every 12 hours for 5 days. The long-term mortality was 53%.

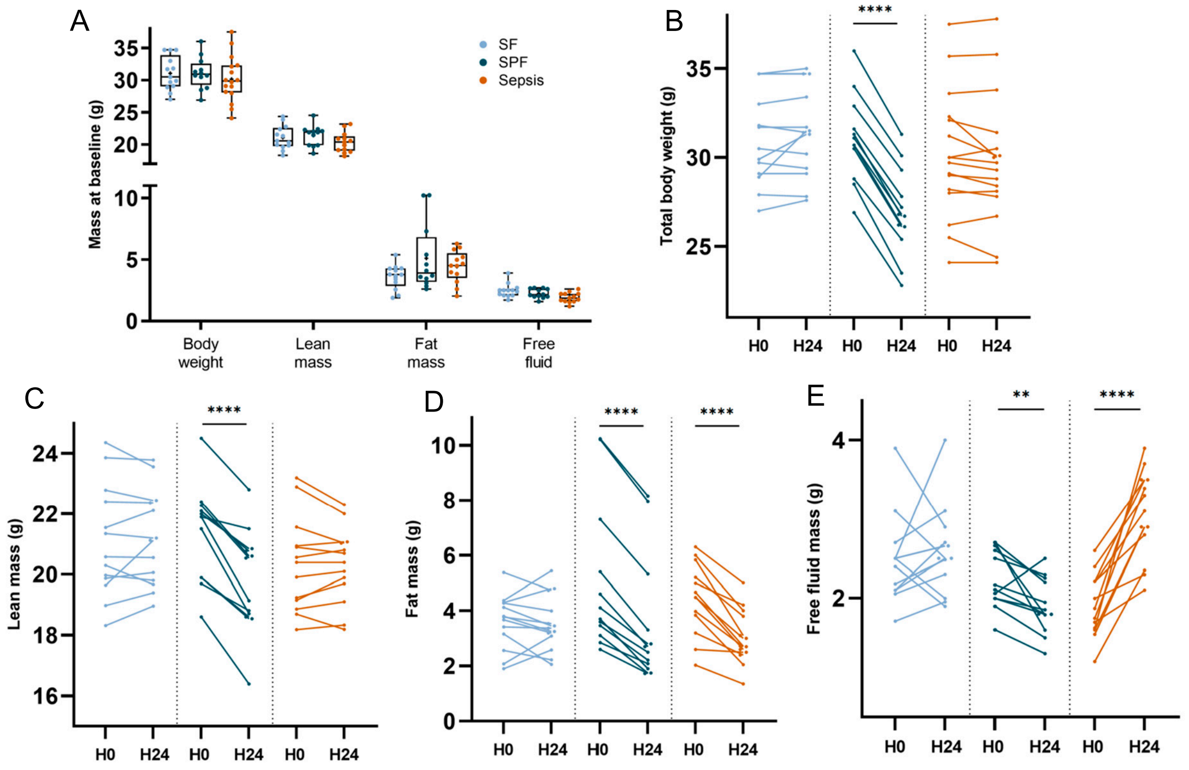

Supplementary Figure S2. Raw data of physiological parameters at baseline and at the 24th hour. (A) Body weight, lean mass, fat mass, and free fluid mass were not different between groups (Sham-fed (SF) *vs.* Sham pair-fed (SPF) or SPF *vs.* Sepsis) at baseline. **(B-E)** Individual changes between H0 and H24 of body weight, lean mass, fat mass, and free fluid mass. The light-blue dot represents the SF group (n=13), the dark-blue dot is SPF (n=12), and the orange dot is Sepsis (n=18 and 13 for body composition). Data from panel A are represented as boxes and whiskers with minimum to maximum and compared between groups with a one-way ANOVA test. Data from panels B to E are represented as scatter dots with connecting lines between H0 and H24 values and these values were compared within a group with a paired-t test. ** $p<0.01$, **** $p<0.0001$.

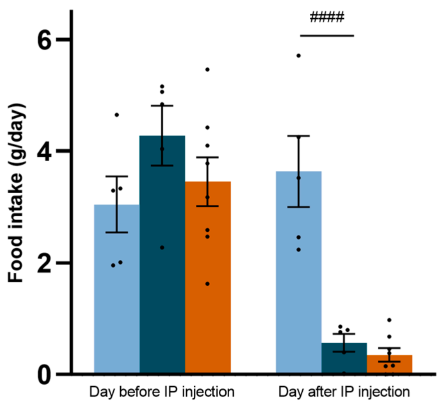

Supplementary Figure S3. Food intake during the metabolic experiment. The day before or after the intra-peritoneal (IP) injection refers to the 24 hours before or after the IP injection of PBS-glycerol or cecal slurry. Daily food intake (expressed in gram of pellets per day) was not different between groups before the IP injection and was drastically reduced in SPF and Sepsis group after IP injection. The diet (U8220G10R, SAFE, France) contained 3,3 kcal/g food, 19.3% proteins, 8.4% fat, 72.4% carbohydrate. Light-blue bars for the SF group (n=5), dark-blue bars for SPF (n=5), and orange bars for sepsis (n=8). Data are expressed as mean values with SEM and compared using a Kruskal-Wallis test with post-hoc Dunn's test. # SPF vs. SF and * Sepsis vs. SPF. **** $p<0.0001$.

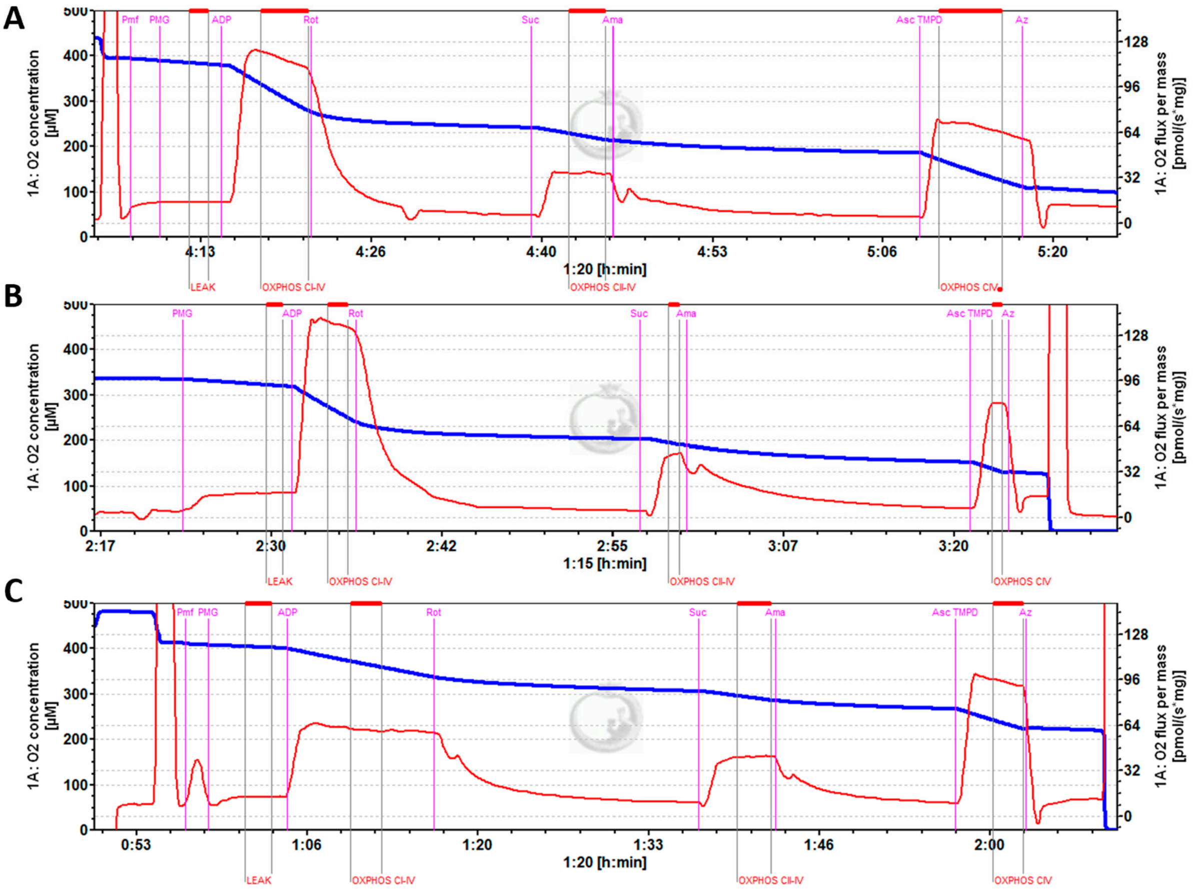

Supplementary Figure S4. Representative oxygraphy curves of *Soleus* permeabilized muscle fibers. Data from **(A)** Sham fed, **(B)** Sham pair-fed, and **(C)** Sepsis mice. The blue line represents the O_2 concentration (µM) and the red line is the oxygen consumption (JO_2) normalized to permeabilized muscle fibers (pmf) mass (pmol.s^{-1}.mg^{-1}). Green or pink lines represent the moment when the chemical product is injected into the chamber. The red frame represents the measurement of the steady state. Pmf of Soleus are incubated in the oxygraphy chamber and JO_2 is recorded after sequential injections: pyruvate (5 mM) malate (2 mM) and glutamate (10 mM) (PMG) (LEAK); ADP (5 mM) (CI-IV for oxidative phosphorylation (OXPHOS) state driven by complex I); rotenone (Rot, 0.5 µM) and succinate (Suc, 10 mM) (CII-IV for OXPHOS state driven by complex II); antimycin A (Ama, 2.5 µM), ascorbate (Asc, 2 mM) and TMPD (0.5 mM) (CIV for OXPHOS state driven by CIV).

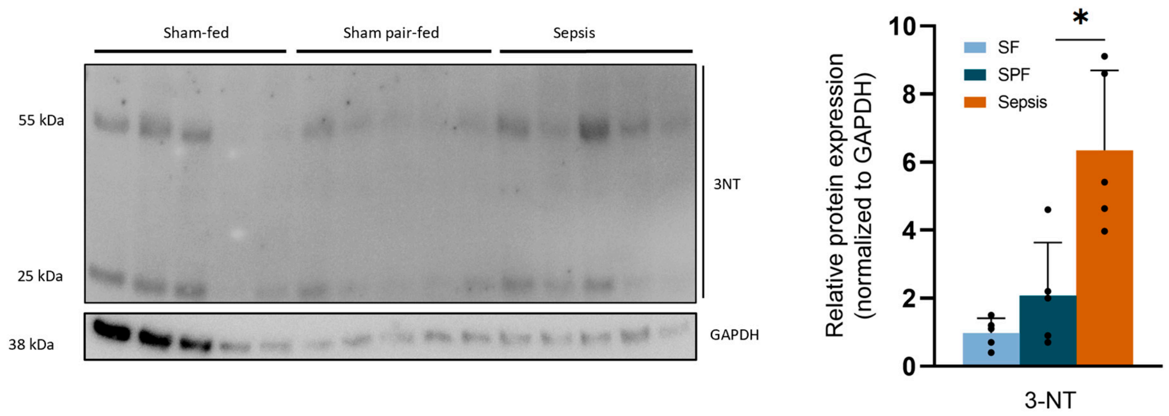

Supplementary Figure S5. Sepsis induced nitrosative stress in skeletal muscle. Relative protein expression of 3-nitrotyrosine proteins was threefold higher in the Sepsis group compared with the SPF group (p=0.06). Light-blue bars for the SF mice, dark-blue bars for SPF mice, and orange bars for Sepsis mice (n=5 per group). Data were expressed as means with SEM and compared using a Kruskal-Wallis test with post-hoc Dunn's test. # SPF *vs.* SF and * Sepsis *vs.* SPF. * $p<0.05$.
